# Prolonged Corticosteroid Use in the Treatment of Tuberculous Meningoencephalitis: A Case Report

**DOI:** 10.3390/medicina61020214

**Published:** 2025-01-25

**Authors:** Annija Holstroma, Arturs Balodis, Artis Brokans, Anda Viksna

**Affiliations:** 1 Faculty of Medicine, Riga Stradins University, 16 Dzirciema Street, LV-1007 Riga, Latvia; 2 Department of Radiology, Riga Stradins University, 16 Dzirciema Street, LV-1007 Riga, Latvia; arturs.balodis@rsu.lv (A.B.); dr.artis.brokans@gmail.com (A.B.); 3 Institute of Diagnostic Radiology, Pauls Stradins Clinical University Hospital, 13 Pilsonu Street, LV-1002 Riga, Latvia; 4 Department of Infectology, Riga Stradins University, 16 Dzirciema Street, LV-1007 Riga, Latvia; anda.viksna@rsu.lv; 5 Centre of Tuberculosis and Lung Diseases, Riga East Clinical University Hospital, LV-2118 Upeslejas, Latvia

**Keywords:** tuberculosis, tuberculous meningoencephalitis, central nervous system, case report

## Abstract

Tuberculous meningoencephalitis is a rare manifestation of *Mycobacterium tuberculosis (Mtb)*, with the most severe form and highest mortality. It can cause multiple complications, and treatment is difficult, as drugs cannot properly diffuse through the haemato-encephalitic barrier. We reported the case of a 17-year-old female patient who was admitted to the emergency room department with a fever for previous two weeks (up to 39 °C), dizziness, difficulty walking, and weight loss. Magnetic resonance imaging indicated possible meningoencephalitis, and a CT scan of the lungs visualised miliary infiltrates in both lungs. After repeated tests, *Mtb* DNA was found in the bronchial wash, cerebrospinal fluid, faeces, and urine via an Xpert/Rif Ultra test. Treatment was started with isoniazid, rifampicin, ethambutol, pyrazinamide, and corticosteroids as well. Although treatment was initiated within the first few days in the hospital, a reduction in glucocorticoid dosage worsened the patient’s neurological state, making treatment even more challenging. Prolonged use of glucocorticoids led to an improvement in the stage of the condition. Further, over time, the patient’s condition improved. Pulmonary infiltrations were not found after 2.5 months of starting therapy. Conclusions: Timely treatment is crucial for improving the prognosis of patients with miliary tuberculosis and tuberculous meningoencephalitis. Prompt recognition of symptoms and accurate diagnosis are essential to initiate effective treatment strategies. In this patient’s case, prolonged use of corticosteroids reduced neurologic complications, and ongoing treatment gradually improved the patient’s condition.

## 1. Background

Tuberculosis (TB) is still a global public health burden. A global estimate of 10.8 million people (95% uncertainty interval [UI] 10.1–11.7 million) had TB in 2023, and 1.25 million died the same year [1]. Tuberculosis is likely, once again, the foremost cause of death globally from a single infectious agent [1].

**Figure 1:**
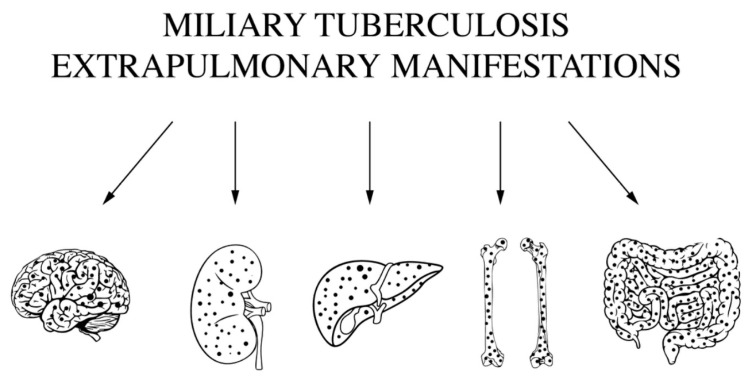
Most common extrapulmonary manifestation sites for miliary tuberculosis. Miliary tuberculosis most commonly affects organs with high blood flow.

Miliary TB is a hematogenous dissemination of *Mycobacterium tuberculosis (Mtb)*, with 1–2 mm wide infiltrations, with extrapulmonary manifestations that often affect organs with high blood flow, e.g., the kidneys, liver, spleen, adrenals, and bone marrow, as well as the central nervous system (CNS) (Figure 1) [2,3]. Dissemination in CNS is relatively rare, in 1–5%, of cases; however, with the most severe form and highest mortality for adults, this increases to 50% [4]. At-risk patients include patients with compromised immune systems, such as those with HIV, diabetes mellitus, or regular steroid use, alcoholism, malnutrition, or chronic kidney failure [3].

Symptoms are usually non-specific, commonly presenting with a fever, weight loss, fatigue, and headache [5]. Acute-stage symptoms are difficult to differentiate from other bacterial or viral meningitis agents, and diagnosis is challenging, as cerebrospinal fluid (CSF) analysis could contain small amounts of Mtb DNA, and repeated tests may be needed for early diagnosis and better patient outcomes [4]. TB as a diagnosis should be considered if CSF analysis shows high protein levels, low glucose levels, and lymphocytic pleocytosis, which differs from bacterial and viral meningitis [6]. Bacterial meningitis CSF analysis, similar to Mtb meningitis, has an elevation in total protein and lower glucose levels; however, these can be mild-to-marked, and have increased total white blood cell counts with higher neutrophil levels [6]. In comparison, typical findings in viral meningitis CSF assessment usually present normal glucose and mild-to-marked protein levels with lymphocyte predominance [6]. Therefore, clinicians should remain vigilant, and treatment should commence promptly upon suspicion of the diagnosis.

When timely initiation of therapy is crucial and CSF data do not indicate TB, the interpretation of radiological examinations plays a significant role.

The computed tomography (CT) and magnetic resonance imaging (MRI) findings in neurotuberculosis, particularly in cases involving caseating tuberculomas, often reveal distinctive patterns that are instrumental in differentiating these lesions from other types of intracranial abnormalities. In patients with caseating neurotuberculosis, tuberculomas usually show up as hypoattenuated (low-density) lesions on non-contrast CT scans [7]. Smaller lesions can be difficult to detect without contrast since there is no ring enhancement, which is frequently seen with contrast. Even on a non-contrast scan, surrounding oedema may help locate these masses by appearing as a faint, low-density areas surrounding the lesions [8]. An MRI provides more detailed information, displaying variations that correspond to the specific stage of the lesion. On T1-weighted images, solid caseating tuberculomas typically appear isointense or hypointense, while on T2-weighted images, they present as hypointense with a surrounding hyperintense rim due to oedema. The high protein content and dense caseous material in the core of the lesion are the main causes of this T2 hypointensity, which lowers the T2 signal. In contrast to other lesions that may exhibit core hyperintensity on T2-weighted images as a result of liquid necrosis, tuberculomas with solid caseous centres appear black in T2 imaging [8]. Only a few pathologies can present with a hypointense signal on T2-weighted MRI sequences, which is due to low tissue water content and increased density, with inflammatory granulomatous process being one of these, such as tuberculosis, as well as, more rarely, fungal infections in differential diagnoses [9]. A key feature in imaging caseating tuberculomas is the rim enhancement observed on post-contrast T1-weighted images. This effect typically arises when an inflammatory zone surrounds a necrotic core within the lesion. Peripheral enhancement occurs due to the accumulation of contrast agents in the viable, actively inflamed tissue surrounding the necrotic centre, highlighting the granulomatous inflammation. This pattern aids in distinguishing tuberculomas from other brain diseases, such as metastases or gliomas, which may have a distinct pattern of enhancement or more uniform enhancement without a rim [10,11].

Glucocorticoid (GC) usage in miliary TB of the CNS is vital, as it may reduce inflammation, oedema, inflammation of small blood vessels, and intracranial pressure [12]. Studies show that while it lowers the risk of death, there is no significant advantage when it comes to severe disability, as it primarily diminishes life-threatening conditions [12,13]. With its known benefits and concerns in patient outcomes, the recommended duration of GC therapy is 6 to 8 weeks with TB meningitis [14]. Close patient monitoring is necessary to prevent GC therapy-associated complications, such as hyperglycaemia, gastrointestinal haemorrhage, immunosuppression, electrolyte imbalance, and hypertension [12].

This clinical case aimed to demonstrate that extended corticosteroid therapy in miliary tuberculosis with meningoencephalitis provided superior clinical benefits, including enhanced disease management and reduced complication rates.

## 2. Case Report

A 17-year-old female patient was admitted to Children’s Clinical University Hospital in Latvia with a fever of up to 39 °C, dizziness, nausea, and difficulty walking, and who had additionally lost 10 kg over the previous months. Previous laboratory tests performed two months prior found elevated C-reactive protein (CRP) levels, at 6.8 mg/L, with no other abnormalities. Laboratory tests performed at the time of admission showed anaemia with a haemoglobin level of 10.5 g/L, elevated alanine aminotransferase, at 83.49 U/L, and elevated CRP, at 7.17 mg/L (Table 1). The patient did not have any chronic diseases and did not take any medications.

**Figure 2:**
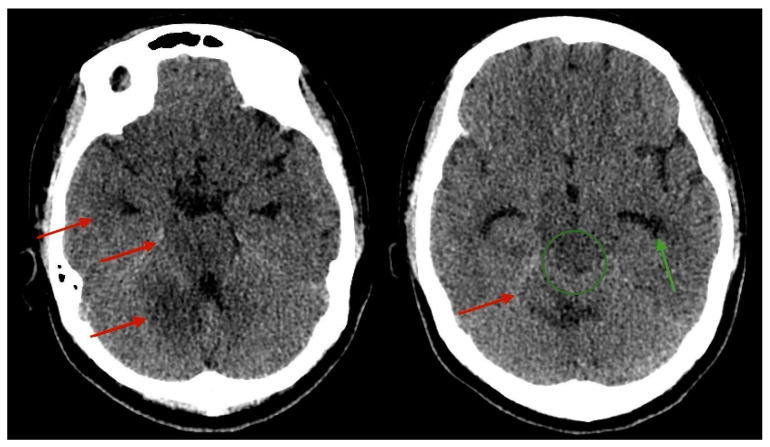
Axial CT scan of the brain reveals hypodense oedematous lesions surrounding the right temporal lobe, brainstem, mesencephalon, pons, and right cerebellum (indicated by red arrows). There is evident dilatation of the temporal horn, measuring approximately 7 mm (green arrow), along with compression of the cerebral aqueduct (green circle), consistent with obstructive hydrocephalus.

A head CT scan was performed at the time of admission, which revealed hypodense changes surrounding the right temporal lobe, mesencephalon, pons, and right cerebellum, with dilatation of the temporal horn and compression of the cerebral aqueduct also visible (Figure 2).

**Figure 3:**
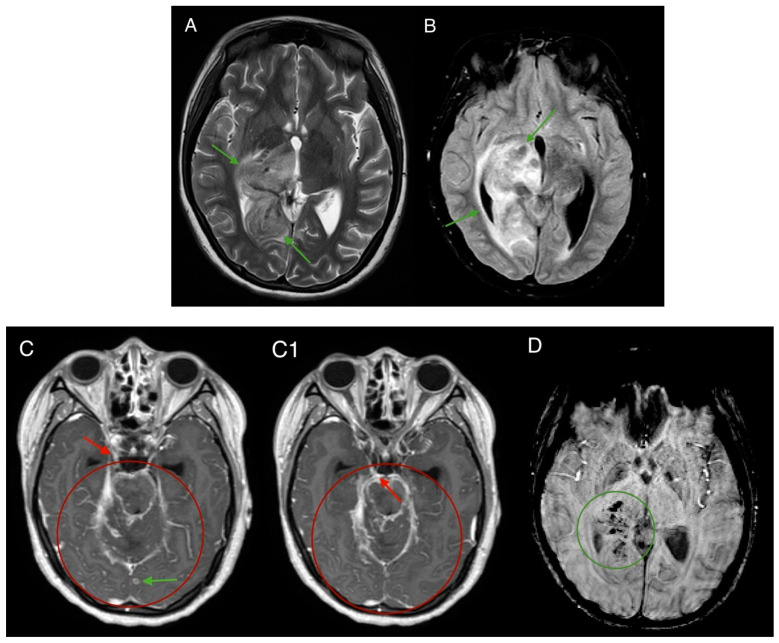
MRI brain scan—T2 (**A**) and FLAIR (**B**) sequences, axial plane: Oedema is observed in the medial portion of the right temporal lobe, as well as in the right occipital lobe, right parietal lobe, mesencephalon, and thalamus, and around the right cerebellum (green arrows). Consistent with previous findings, evidence of obstructive hydrocephalus is also present. MRI brain scan-T1 post-cariscan contrast sequence (**C**,**C1**), axial plane: Post-contrast enhancement is observed around the right cerebellar peduncle, mesencephalon, pons, and basal cisterns, with the findings suggestive of leptomeningitis (red circle). Additionally, enhancement around intracranial nerves is visible (red arrows—oculomotor nerve). Approximately twenty small nodules, each measuring 1–2 mm with peripheral contrast enhancement, are distributed throughout the brain (green arrow). MRI brain scan-T2 SWI sequence (**D**), axial plane: Accumulation of blood product deposits is noted within the oedematous regions (green circle).

A head MRI scan found oedematous changes with hemosiderin deposition in the right hemisphere, including the thalamus, temporal lobe, parietal lobe, mesencephalon, and right cerebellum, suggesting meningoencephalitis and basal cisterns with leptomeningeal enhancement (Figure 3). Initial treatment before bacteriology results came back was started with 500 mg of acyclovir and 2000 mg of ceftriaxone.

Lumbar puncture was performed, and an assessment of the CSF found a high protein level at 2.15 g/L (Ref. 0.15–0.45) and low glucose levels—1.11 mmol/L (Ref. 2.2–3.89). However, no *Mtb* DNA, nor any other bacteriological agent, was found in the sample.

**Figure 4:**
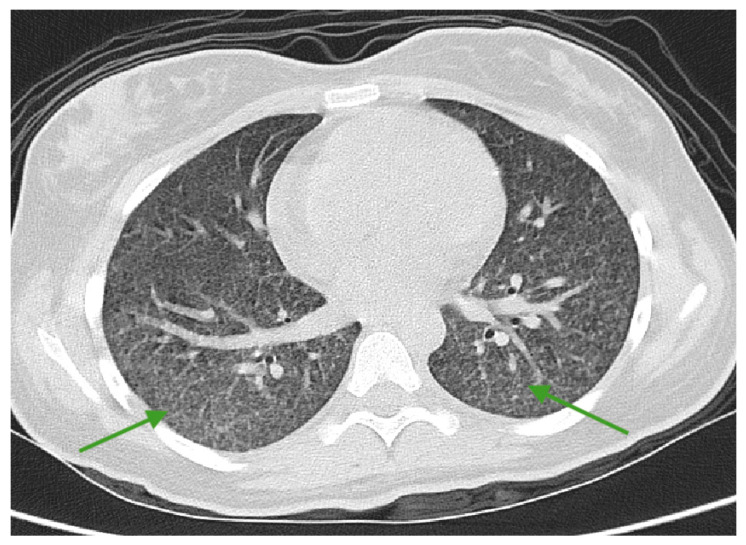
CT scan of the lungs and axial plane: Diffusely uneven pulmonary pneumatization with prominent micronodular dissemination can be seen all over both lungs. Denser clusters of dissemination are noted in the posterior segments of the lower lobes (green arrows).

Control chest CT scan with intravenous contrast showed miliary infiltrates in the lungs (Figure 4). Bronchial wash analysis found Mtb DNA, and additionally, it was detected in the second CSF analysis at a very low concentration using the Xpert MTB/Rif Ultra test, and confirming the diagnosis of miliary pulmonary tuberculosis with tuberculous meningoencephalitis (Table 2). Use of the Xpert MTB/Rif Ultra test additionally revealed that Mtb DNA levels were at a very low concentration in the faeces, and was “trace” positive in urine tests; however, gastrointestinal or genitourinary system involvement was not found and was detected due to the Mycobacterium shedding phenomenon.

On the fourth day of admission to the hospital, anti-tuberculous treatment was started following WHO guidelines for drug-susceptible *Mtb* with 300 mg of isoniazid, 600 mg of rifampicin, 1000 mg of ethambutol, and 1500 mg of pyrazinamide; the dosage was calculated in line with the patient’s weight. Antituberculous therapy was performed in combination with 12 mg of dexamethasone as the initial dose.

During the first week of treatment, the patient was admitted to the paediatric intensive care unit (ICU) due to severity of state.

After a month of therapy, complications associated with CNS tuberculosis developed: seizures, coma, obstructive hydrocephalus, acute ischemia in the left parietal lobe with acute paresis, and asymmetrical tetraparesis, and the patient was readmitted to the ICU. The patient underwent a tracheostomy following mechanical ventilation for more than 10 days, and percutaneous endoscopic gastrostomy was performed as well.

**Figure 5:**
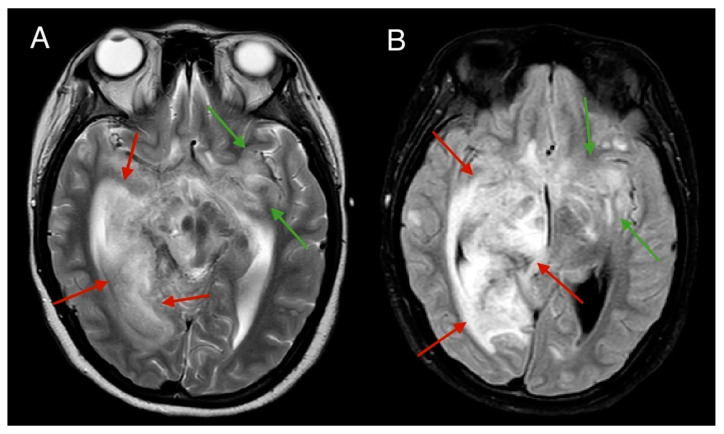
MRI scan of the brain, T2 (**A**), FLAIR (**B**) sequences, and axial plane. Progressive MRI findings: Around one month after the initial scans, MRI findings demonstrate worsening changes. Progressive oedema is observed around the temporal and parietal lobes, mesencephalon, thalamus, and right cerebellum (red arrows), with extension into the left temporal and frontal lobes (green arrows). Compression of the lateral and third ventricles can also be observed, accompanied by a dynamically worsening midline shift.

In the control MRI scan, progressive oedema was noted in the temporal and parietal lobes, mesencephalon, thalamus, and right cerebellum, extending into the left temporal and frontal lobes. Additionally, features of obstructive hydrocephalus were observed. (Figure 5). A ventriculoperitoneal shunt was placed. Furthermore, a worsened neurological condition correlated with a decreased dosage of dexamethasone, which was gradually reduced from 12 to 4 mg, as the patient became unresponsive and experienced repeated seizures.

**Figure 6:**
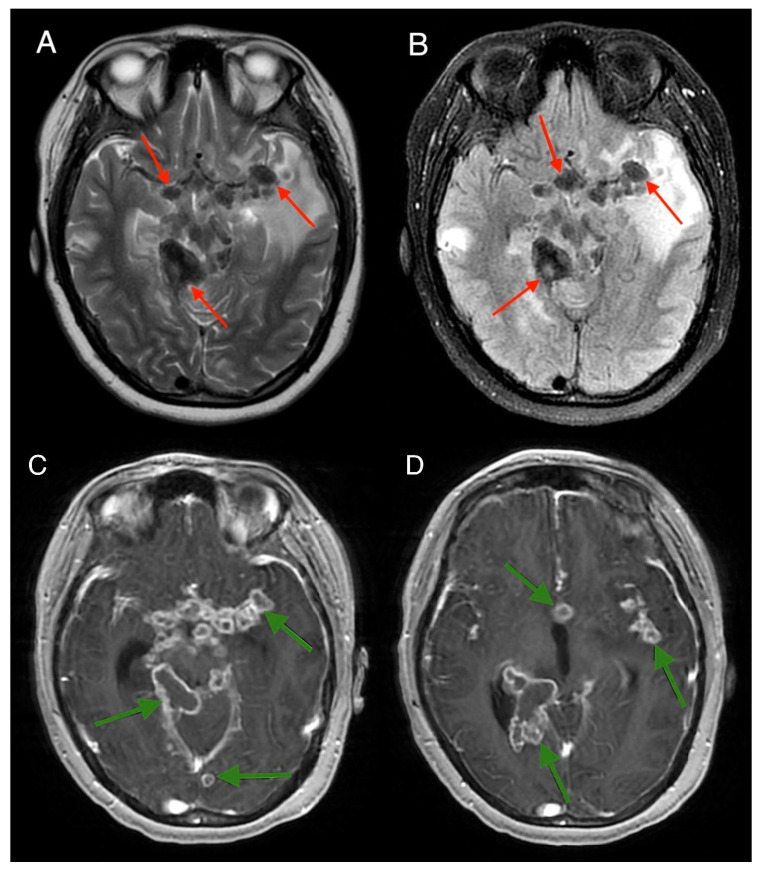
MRI brain scan—axial plane, T2 (**A**), and FLAIR (**B**) sequences: Extensive spread of hypointense tuberculous granulomas (caseating) is observed, predominantly around the brainstem, basal cisterns, circulus arteriosus (circle of Willis), and mesencephalon (red arrows). Oedema remains evident in both sequences. MRI brain scan—T1 post-clariscan sequences (**C**,**D**), axial plane: Diffuse granulomatous tuberculous leptomeningitis is demonstrated, with extensive spread of tuberculous granulomas (green arrows) primarily surrounding the brain stem, basal cisterns, circle of Willis, and mesencephalon. The tuberculomas exhibit a caseating granulomatous appearance with peripheral contrast enhancement following contrast administration.

After 2.5 months of therapy, the patient was transferred to the Centre of Tuberculosis and Lung Diseases in Riga East University Hospital, Latvia. The patient’s condition remained severe, with spontaneous breathing through the tracheostomy and continued inability to swallow food. A control MRI T2 sequence found an extensive dissemination of hypointense, caseating tuberculous granulomas, primarily involving the brainstem, basal cisterns, circle of Willis, and mesencephalon (Figure 6). Chest X-ray imaging did not find miliary infiltrates.

After 5.5 months, the patient regained consciousness and gradually began communicating. The patient could walk with assistance after physiotherapy. A control head MRI scan after 10 months showed a decreased size of tuberculomas, and no additional structural changes were found.

During treatment, pyrazinamide was discontinued after 3 months of therapy. At the same time, isoniazid was changed to levofloxacin with a dosage of 1000 mg due to non-convulsive seizure episodes. Dexamethasone was continued for 9 months. The decision to continue dexamethasone for 9 months during TB treatment was crucial as the patient’s neurological status deteriorated when the dexamethasone dosage was reduced.

## 3. Discussion

The rate of global TB incidence has been gradually increasing since the COVID-19 pandemic, and the incidence of miliary TB is expected to rise relatively due to increased usage of immunosuppressive drugs and HIV. Miliary TB represents a significant health concern, comprising around 1–2% of all tuberculosis cases and 8% of those that are extrapulmonary [15]. TB treatment in Latvia has been outlined by World Health Organization guidelines since 1996. Over time, the incidence of miliary TB decreased in Latvia; there were 6 cases in 880 total TB patients in 2012, and 2 cases in 560 TB patients in 2016, and of those, CNS TB was only present in 3 [16].

Patients at risk of miliary tuberculosis include young children, those who have diabetes, those with immunosuppression (including the use of biological and other immunosuppressive drugs), those with HIV infection, chronic haemodialysis patients, smokers, or those with other underlying diseases [17,18]. In our case, we demonstrated that the patient was previously healthy and with no other predisposing risk factors that could predetermine miliary TB infection with manifestations in the CNS.

Bacteriology tests are commonly repeated multiple times, as CNS TB CSF has a low bacillary load, making diagnosis difficult [19]. It is practical to obtain larger volumes of CSF (~10 mL) in cases where CNS TB is suspected based on the patient’s length of symptoms and imaging test results [20]. Another way to successfully detect *Mtb* in CSF is sample centrifugation, but data for this still need to be clarified. A study performed by Patel et al. shows data highlighting that sensitivity and specificity were 94% for centrifuged vs. 35% for uncentrifuged CSF Xpert MTB/Rif tests, but only with people living with HIV Mtb meningitis [21]. A case report published by Ghimire et al. similarly found the diagnosis of tuberculous meningitis challenging, and treatment was started based on CSF analysis results, even though the Xpert MTB/Rif Ultra test was negative, as early treatment decreases the risk of complications [22]. In our case, the diagnosis was delayed, and the patient at the time of admission to the hospital had severe progression.

Dexamethasone treatment following WHO guidelines is recommended for 8 weeks as it reduces the mortality rate [23]; however, in this case, dexamethasone was administered for 9 months, as each time it was discontinued, the patient developed more severe symptoms and was not communicable. However, Chen et al. emphasise the risk of early osteoarthritis and other complications caused by long-term usage of dexamethasone [24]. It is crucial to closely track the patient’s clinical status and adapt the therapy in response to their symptoms.

While imaging tests suggested meningitis for our patient, it was crucial to keep in mind the distinction between TB meningitis and other forms of meningitis. For later stages of the disease, a key feature in imaging caseating tuberculomas is the rim enhancement observed on post-contrast T1-weighted images. This effect typically arises when an inflammatory zone surrounds a necrotic core within the lesion. Peripheral enhancement occurs due to the accumulation of contrast agents in the viable, actively inflamed tissue surrounding the necrotic centre, highlighting the granulomatous inflammation. This pattern aids in distinguishing tuberculomas from other brain diseases, such as metastases or gliomas, which may have a distinct pattern of enhancement or more uniform enhancement without a rim [10,11]. Study conducted by Ma. et al. found that tuberculomas were detected for all patients through contrast-enhanced T1-weighted images, with most of them presenting as mildly hypointense and almost indistinguishable from normal cerebral grey mater; more than half of patients on T2-weighted and FLAIR images demonstrated a hypointense core with a hyperintense rim, while others had signs of mild hyperintensity [10].

We present this case as it is essential to examine the patient to find the right diagnosis and, therefore, the best treatment to reduce complications and mortality risk. The presented diagnosis was not easily achieved. It is important to perform tests multiple times, and even a simple imaging test of the chest could either confirm or dismiss a possible diagnosis.

## 4. Conclusions

Miliary tuberculosis can be a challenge to diagnose and verify through laboratory and microbiological findings; therefore, repeated tests are necessary, delaying timely administration of antituberculous therapy. MRI has a vital part as an early diagnostics tool, while we recognise the importance of key features for tuberculous granulomas in later stages of the disease. Furthermore, each patient has to have an individualised approach to allow for the best therapy to be chosen. In this case, dexamethasone was used for a longer period than the guidelines recommended, as it improved the patient’s condition.

## Figures and Tables

**Table 1:** Laboratory tests at the time of admission with anaemia, elevated aminotransferase, and CRP. (ALAT—alanine aminotransferase, APTT—activated partial thromboplastin time, CRP—C-reactive protein, Er—erythrocytes, GFR—glomerular filtration rate; Hct—haematocrit, Hgb—haemoglobin, INR—international normalised ratio, Leu—leukocytes, Tr—platelets).

Haematology	Results	Reference Value
Leu, ×10^9^	8.88	4–10
Er, ×10^12^	4.39	4.2–5.4
Hgb, g/L	10.5	12.0–16.0
Hct,%	30.1	37–47
Tr, ×10^9^	275	150–400
Hemostasis		
INR	1.04	0.8–1.2
D-dimer	3.11	0–0.5
APTT, s	28.8	28–40
Biochemistry		
ALAT, U/L	83.49	10–49
Glucose, mmol/L	7.24	4.1–5.9
Urea, mmol/L	4.46	3.2–8.2
Creatinine, umol/L	52.17	49–90
GFR, ml/min	135	
Sodium, mmol/L	128.79	136–145
Potassium, mmol/L	4.46	3.5–5.1
CRP, mg/L	7.17	0–5

**Table 2:** Bacteriology tests with sample sites and dates (CSF—cerebrospinal fluid, RIF—rifampicin).

Date	Sample	Direct Fluorescence Antibody Test	Xpert MTB/Rif Ultra Test	Bactec Medium	Löwenstein—Jensen Medium
1 April	CSF	Negative	Negative	Positive	-
5 April	Blood	-	-	Negative	-
6 April	CSF	Negative	Positive, RIF-sensitive	Negative	Negative
6 April	Urine	Negative	“Trace”	Negative	Negative
7 April	Bronchial wash	Negative	Positive, RIF-sensitive	Positive	Positive
8 April	faeces	Negative	Positive, RIF-sensitive	Negative	Negative

## Data Availability

The original contributions presented in this study are included in the article. Further inquiries can be directed to the corresponding author.
